# Honeybees control the gas permeability of brood and honey cappings

**DOI:** 10.1016/j.isci.2022.105445

**Published:** 2022-10-27

**Authors:** Jiří Kubásek, Karolína Svobodová, František Půta, Alena Bruce Krejčí

**Affiliations:** 1 University of South Bohemia, Faculty of Science, Ceske Budejovice, Czech Republic; 2 Department of Cell Biology, Faculty of Science, Charles University, Prague, Czech Republic; 3 Czech Academy of Sciences, Biology Centre, Institute of Entomology, Ceske Budejovice, Czech Republic

**Keywords:** Biochemistry, Biophysics, Biological sciences

## Abstract

Some bee species use wax to build their nests. They store honey and raise their brood in cells made entirely from wax. How can the bee brood breathe and develop properly when sealed in wax cells? We compared the chemical composition and structural properties of the honey cappings and worker brood cappings of the honeybee *Apis mellifera carnica*, measured the worker brood respiration, and calculated the CO_2_ gradients across the two types of cappings. We identified microscopic pores present in the brood cappings that allow efficient gas exchange of the developing brood. In contrary, honey cappings are nearly gas impermeable to protect honey from fermenting. Similar principles apply in bumble bees. Our data suggest the control of gas exchange of cappings as a selective pressure in the evolution of wax-building bees that drives their adaptation for using wax in two highly contrasting biological contexts.

## Highlights

• Brood cappings are well permeable to CO_2_, similarly to living cells and organs
• Brood cappings, but not honey cappings, contain pores
• Honey cappings are poorly permeable to CO_2_, as apical membrane of gut cells
• Permeability of cappings as a selective pressure in the evolution of wax-building bees

## Graphical abstract

**Figure:**
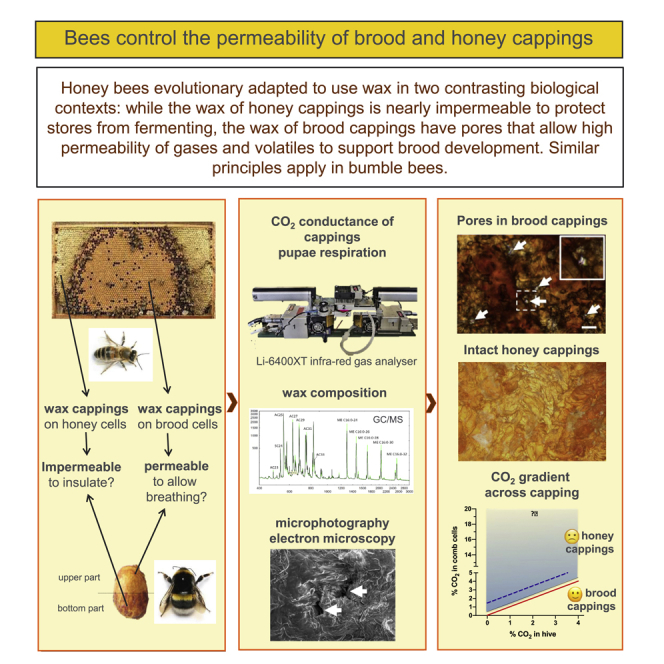

## Introduction

Honeybees (*Apis*), stingless bees (*Meliponini*), and bumble bees (*Bombus*) are the only known insects that have evolved the ability to use wax for building their nests. The wax cells in the nest, often associated in combs, serve for storing nectar, honey and pollen resources as well as for raising brood, besides their function as a humidity and temperature buffer^1^^,^^2^ as a communication device via vibrational and smell cues^3^^,^^4^ and as place for the bees to gather.^5^ The use of wax cells closed with cell cappings allowed these species to store food for long periods of time, providing an evolutionary advantage to them over other bee species.

Individual wax cells can be used for storage or for rearing brood, depending on the actual needs of the colony. Once an egg hatches within a cell, the honeybee larva is incubated and fed a specific diet by the nurse bees for a species defined period of time.^6^^,^^7^ The brood cell is then capped by the workers,^8^ permitting the larva to spin its cocoon, transform into a pupa and complete its development inside the comb cell.^9^ Bumble bees can rear several larvae together in one wax cell at the beginning of their development. The larvae of stingless bees are not fed by the adults because the cell is capped straight after egg deposition and the hatched larva utilizes pollen and honey stores deposited within the cell. Similarly, nectar is also stored in the wax cells where it is enriched and concentrated by the bees. Moreover, honeybees and stingless bees are able to store ripe honey in comb cells for several months, but only after coating them with wax cappings.^10^

The wax used to construct comb cells and their cappings is composed of more than 300 constituents, including fatty acid esters, hydrocarbons and free fatty acids.^11^^,^^12^ Its lipophilic nature is well suited for storing of aqueous solutions such as honey. Similar lipidic mixtures present in insect or plant cuticles are also very impermeable to gases and water vapor.^13^ Thus, wax cappings formed on ripe honey cell stores offer good protection against water reabsorption that could lead to honey fermentation because of the ubiquitously present yeast from the environment.^14^ This can be considered as an important evolutionary adaptation that allows the bees to overcome long dearth periods when pollen and nectar sources are limited or absent.

Covering brood with wax capping is beneficial for proper brood development in the hive conditions by helping to buffer the humidity or temperature conditions.^1^^,^^15^ *In vitro*, the honeybee pupa can develop normally without capping as long as correct temperature and highly humid conditions are maintained.^16^ Within the context of the comb, the porosity and rough texture of the brood cappings serve as an important cue that helps to orient the larva longitudinally in the cell after spinning its cocoon, so that its head lies toward the capping and allows hatching later.^17^ The importance of brood capping is also illustrated by the fact that opened brood at the stage of pupae is rarely seen in honeybee colonies, except for a short time during the recapping process^18^ or during the hygienic behavior when unhealthy pupae are removed from the comb.^19^

Applying the impermeable properties of honey cappings to the wax cappings of brood cells would limit the gas exchange associated with the breathing of brood. Moreover, permeability of brood cappings is essential for the detection of volatile compounds released by varroa mites that trigger the removal of parasitized brood by the worker bees (varroa sensitive hygiene, VSH).^20^ Therefore, the brood cell cappings must be much more permeable than any judgment solely inferred from the chemical properties of their constituent wax. However, although this fact can be inferred as an obvious concept, there is an extreme paucity in our collective knowledge about how such permeability is achieved. For example, no data on the diffusive conductivity of brood cappings is available in the current literature, meaning gradients of CO_2_, oxygen, water vapor or other volatiles across brood cell cappings cannot be modeled at present.

To elucidate the enigma of the brood capping permeability, we have compared the chemical and structural differences between the worker brood wax cappings and wax honey cappings of the honeybee *Apis mellifera* using GC/MS analysis, microphotography and scanning electron microscopy. We have also determined the diffusive conductances of both types of the cappings by a tandem of infrared gas analyzers and measured brood respiration. Collectively, these datasets allowed us to calculate the expected CO_2_ gradients across the cappings. We discuss the consequences of these differential gradients and their probable significance in instigating evolutionary adaptive behavior in the wax-nest building insects to utilize the same material for two distinction and functionally different purposes.

## Results

### CO_2_ diffusive conductance of the honeybee worker brood cappings and honey cappings

**Figure 1:**
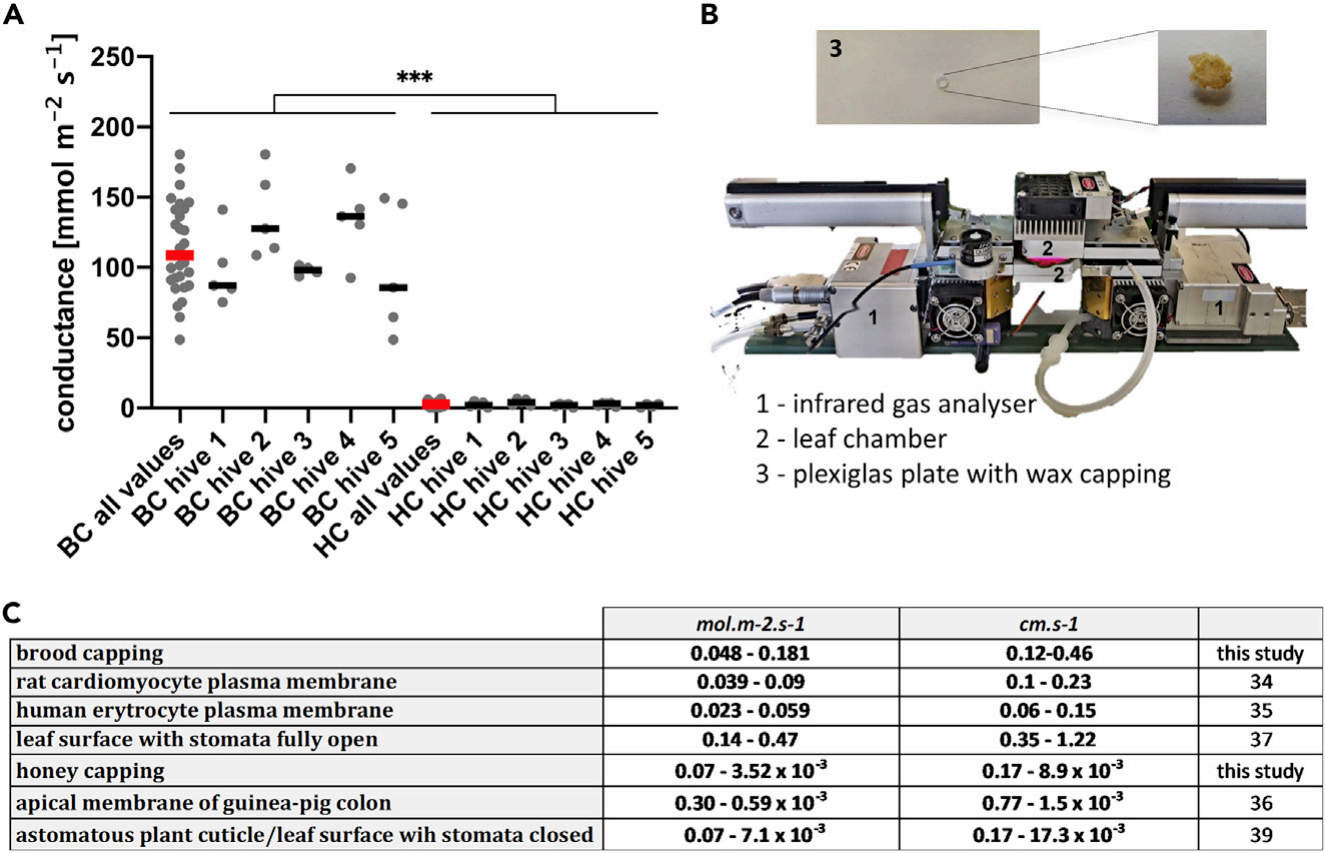
CO_2_diffusive conductance of cappings (A and B) Worker brood cappings 'BC' and honey cappings 'HC' were dissected from the same combs and their conductance for CO_2_ measured by infra-red gas analyzers (5 hives, 5 brood cappings and 5 honey cappings per hive). Individual data points with medians. Mann–Whitney U test (Z = 6.54, p < 0.001). ∗∗∗ indicates p < 0.001 (B) Experimental setup of the tandem of Li-6400XT infra-red gas analyzers. Plexiglass plate with a wax capping is inserted in between the two leaf chambers. (C) Comparison of CO_2_ diffusive conductances of cappings with conductances of various biological systems. See also Figure S1.

The CO_2_ conductance of honey cappings was extremely low, ranging from cca 0 to 5.88 mmol m^−2^ s^−1^, with median 3.3 mmol m^−2^ s^−1^ (n = 25). The lowest values are at the detection limit of the gas analyzer (disadvantaged further by small capping area, about 30-fold lower in comparison to default analyzer chamber area). In contrary, the brood cappings conductance ranged from 64.6 to 180.5 mmol m^−2^ s^−1^, with median 108.7 mmolm^−2^ s^−1^ (n = 25), thus 33-fold higher than the conductance of honey cappings (Figure 1A). This difference is highly significant using Mann–Whitney U test (U = 0, Z = 6.54, p < 0.001).

### Composition and microstructure of the honeybee cappings

We aimed to test the hypothesis that the profound differences in CO_2_ conductance between the cappings could be due to the differences in their chemical composition or their structure.

**Figure 2:**
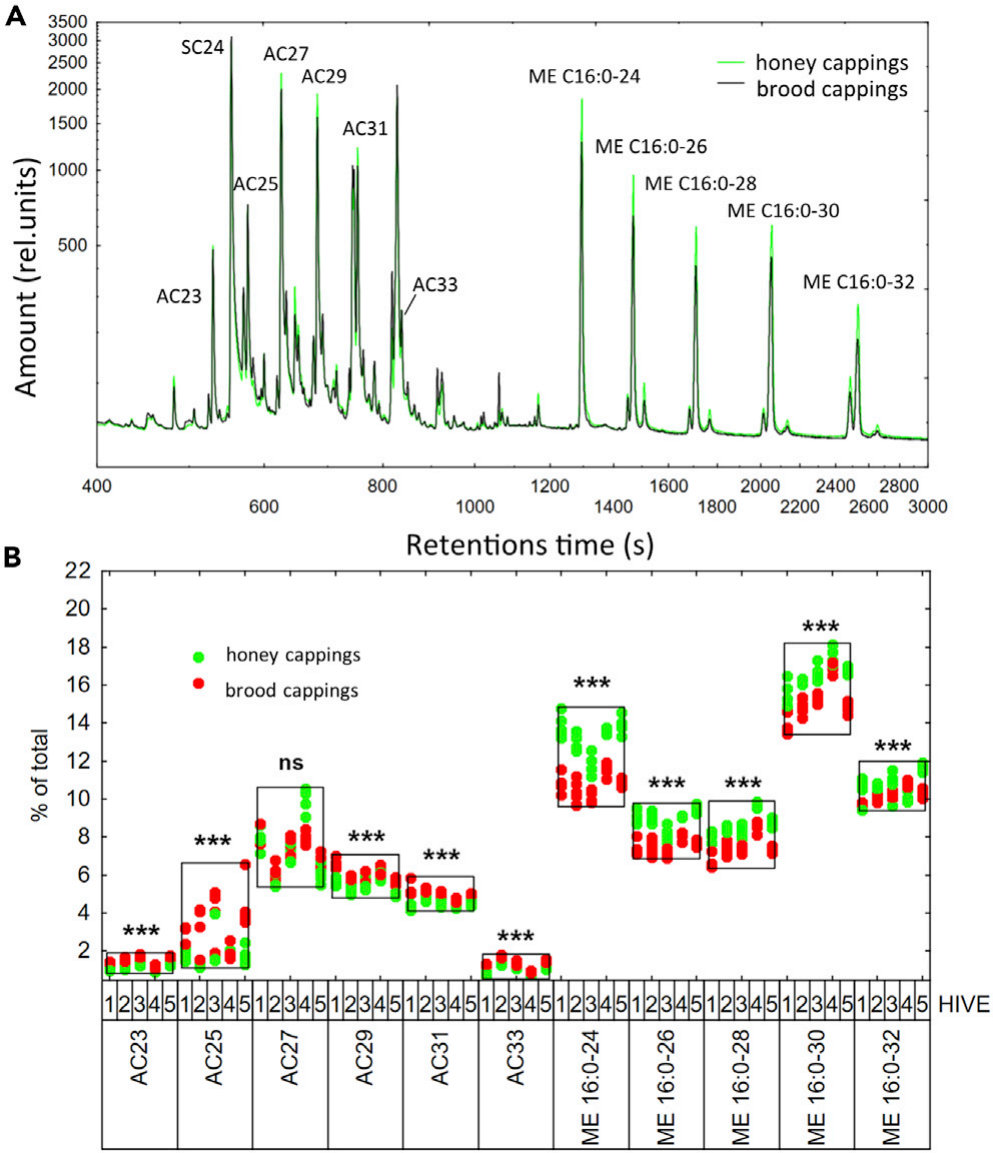
The n-alkane and monoester composition of the cappings Five honey cappings and 5 worker brood cappings each from 5 different hives were dissected from the same combs and their composition assessed by GC-MS. (A) Representative GC-MS chromatograms with individual peaks for n-alkanes (AC), monoesters (ME) and a standard C24 n-alkane (SC24). (B) Quantification of peak sizes for individual n-alkanes and monoesters in honey and brood cappings. N = 25 for each type of cappings. Brood cappings contained lower percentage of monoester content compensated by higher percentage of n-alkanes when compared to the honey cappings (two-way ANOVA F_1, 4_ = 123.8, p < 0.001). The hives did not differ significantly when tested as a random factor (F_4, 23.7_ = 2.20, p = 0.100). ns indicates p > 0.05, ∗∗∗p < 0.001.

Both the honey cappings and brood cappings gave very similar GC/MS profile (Figure 2A), corresponding to the typical profile of honeybee wax.^12^^,^^21^^,^^23^ In agreement with the literature, we identified C27 as the most abundant n-alkane in both types of the cappings, followed by C29, C31 and C25 (Figure 2B). We also quantified palmitates and oleates monoesters that are the most abundant chemical compounds of beeswax.^12^^,^^24^ According to our results, the brood cappings contained lower percentage of monoester content compensated by higher percentage of n-alkanes when compared to the honey cappings (F_1, 4_ = 123.8, p < 0.001, Figure 2B). The hives did not differ significantly when tested as a random factor (F_4, 23.7_ = 2.20, p = 0.100).

**Figure 3:**
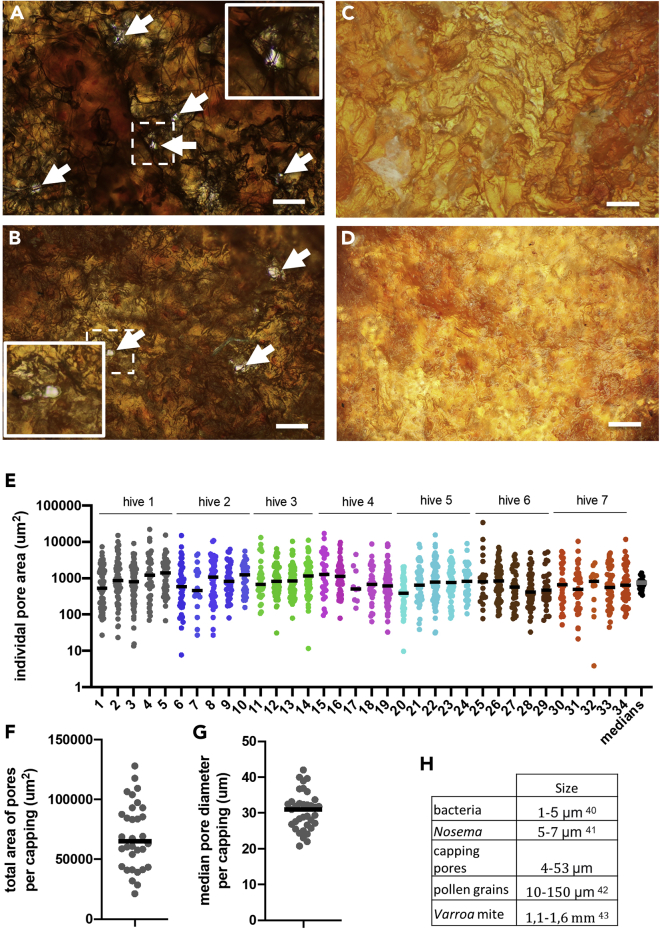
The pores in the worker brood cappings and their quantification Microphotography of (A) inner and (B) outer surface of worker brood cappings and (C) inner and (D) outer surface of honey cappings. Arrows indicates pores throughout the brood cappings. Note the cocoon fibers going across the inner surface in A. Scale bar 100 μm. Insets in A and B show magnified regions indicated by dotted line in the main photograph. (E) Quantification of the pores area per individual cappings across 7 different hives (5 cappings per hive) with median values. Individual values with medians. (F) Quantification of the total area of all pores per capping (pooled data from 7 hives, n = 35 cappings). Individual values with median. (G and H) Quantification of the median pore diameter (pooled data from 7 hives, n = 35 cappings) and (H) its comparison to the sizes of common pathogens and pollen grains found in the hive. See also Figure S1.

**Figure 4:**
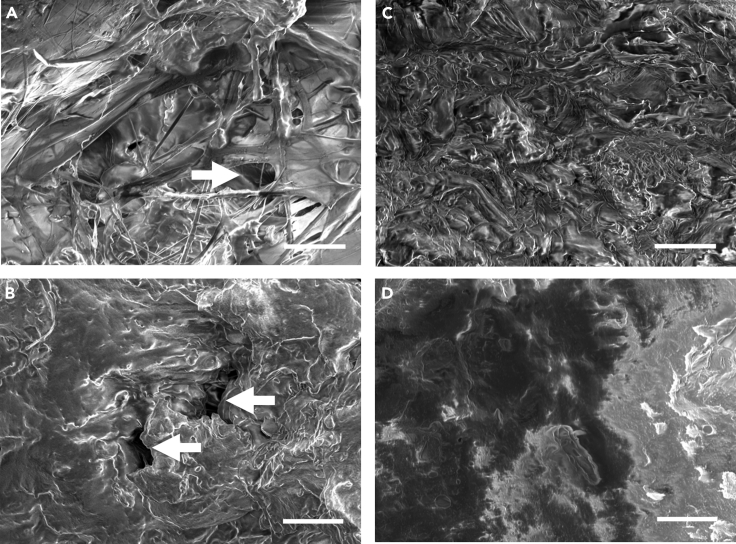
EM pictures of the pores in the worker brood cappings (A–D) Scanning electron microscopy pictures of (A) inner surface of worker brood capping, (B) outer surface of brood capping, (C) inner surface of honey capping, and (D) outer surface of honey capping. Arrows indicate pores in the brood cappings, absent in the honey cappings. Scale bar 50 μm.

At the same time, we found substantial differences in the outer and inner surfaces of both the cappings by optical microphotography and by the scanning electron microscopy. The honey cappings have a smooth surface on their outer side whereas their inner surface is more rough (Figures 3C, 3D, 4C, and 4D). They look as a compact sheet and only very rarely contain visible pores, in agreement with their low CO_2_ conductance. The brood cappings are rough on both their surfaces. As expected, their inner surface is connected with the fibers of the cocoon that the larva spins after capping. Interestingly, there are pores coming across the cappings (Figures 3A, 3B, 4A, and 4B). They are randomly scattered across the capping, often with irregular shapes, and the area of an individual pore covers 753 μm^2^ as a median value (Figure 3E, n = 35 cappings). The corresponding pore diameter spans 4–53 μm, with the median value of 31 μm (Figure 3F). They are larger than the average size of bacterial or fungal cells (e.g. the microsporidian *Nosema*) but they are substantially smaller than the adult parasitic *Varroa* mite (Figure 3F). The median of the total area that is covered by pores is 0.06 mm^2^ per capping, representing 0.29% of the capping surface.

### Respiration rate of developing honeybee pupae

**Figure 5:**
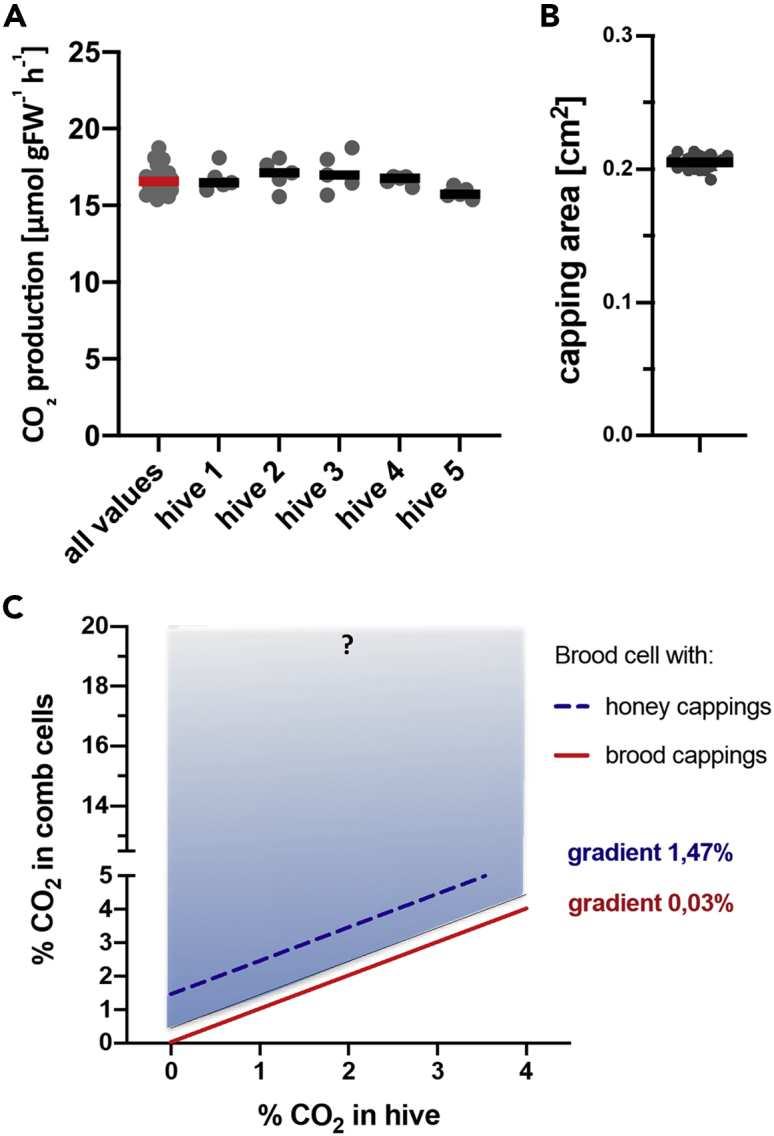
Respiration rate of honeybee pupae, capping area and the expected CO_2_ concentrations in the wax cells with various cappings (A) CO_2_ production (respiration) of honeybee worker pupa, age 10 days since hatching (4 days after cell capping) in 5 different hives, 5 pupae per hive. Individual values with medians. There is no significant difference between the hives (two-way ANOVA F_4,20_ = 2.09, p = 0.120). (B) Capping area of worker brood cell measured by optical microphotography. Individual values with medians (n = 18). (C) The expected CO_2_ gradient of worker brood capping and the expected physiological CO_2_ concentrations in worker brood (red) and CO_2_ gradient and concentrations in a hypothetical situation when worker brood would be covered with honey capping (blue). Blue rectangle shows the span of values for honey cappings based on minimal conductance and maximal pupa respiration and vice versa. The maximal span values of honey capping are likely even bigger, see discussion. On the contrary, the span for brood cappings is very narrow and invisible in the graph. See also Figure S1 and Table S1.

To be able to determine the CO_2_ concentrations in the capped brood cell we first needed to accurately assess the respiration rates of the developing pupae. As it is apparent from Figure 5A, the 10-day-old pupae (4 days after capping) produced 1.98 to 2.69 μmol CO_2_ h^−1^ per pupa (median 2.39, n = 25) that represents respiration rate 13.99 to 18.75 μmol g^−1^FW h^−1^ (median 16.49, n = 25). Data had Gaussian distribution, with low variability. Random effect ‘hive’ was not significant (F_4,20_ = 2.09, p = 0.120).

### Expected CO_2_ gradient across the cappings

Knowing the cappings conductance and the pupae respiration rate we aimed to determine the CO_2_ concentrations inside the worker brood cell where honeybee worker pupae develop. In order to do so we first needed to measure the median area of the cell cappings that is 0.207 cm^2^ (n = 18, Figure 5B). The CO_2_ concentration inside the brood cell depends on CO_2_ concentration in hive and the CO_2_ gradient (that is the difference between the CO_2_ concentration in the hive and in the brood cell) is calculated as a function of pupae respiration rate, capping area and capping diffusive conductance for CO_2_. It will be constant if these parameters do not change. The extremes of respiration rates and capping diffusive conductance, in connection with almost invariable cappings area should define the span of the CO_2_ gradient. According to our measurements, the resulting CO_2_ gradient across the capping should be in a narrow range of 0.01% in case of minimal respiration rate and maximal capping conductance and 0.06% in the opposite contrast (0.03% using both median and average values). In other words, the median or average concentration of CO_2_ in the brood cell will only be 300 ppm (0.03%) higher than the CO_2_ concentrations inside the hive. The brood cappings allow very good exchange of CO_2_ gasses and the CO_2_ concentrations inside the brood cell will never exceed dramatically the CO_2_ concentrations in the hive environment (Figure 5C).

On the other hand, the calculated CO_2_ gradient for a hypothetical situation when the brood would be sealed with cappings from the honey cells lies in a broad range between 0.5 and 15.16% (1.47% in median, 2.81% in average) based on our experimentally determined values but in real situation it could be even higher, see discussion.

### CO_2_ diffusive conductance of bumble bee cocoons and respiration of their pupae

To see the evolutionary conservation of the differential use of wax in the *Hymenoptera* family we compared the characteristics of the honeybee brood cell with the same parameters for the bumblee brood cocoons of *Bombus terretris*.

The cocoon consists of a bottom waxy part and an upper part that contains much less wax (Figure S1A). Inside of the cocoon there are silk fibers spun by the larva before pupation, similarly as in honeybees. The waxy bottom part of the cocoon was nearly impermeable, with the conductance of 0.06–1.5 mmol m^−2^ s^−1^ (median 0.49 mmol m^−2^ s^−1^, n = 10, whereas the upper part was porous with conductance of 2.68–12.72 mmol m^−2^ s^−1^(median 7.4 mmol m^−2^ s^−1^, n = 10). The CO_2_ diffusibility of the upper part of the cocoon was therefore 14.3-fold higher in median values than the CO_2_ diffusibility of the bottom part (U = 0, Z = −3.74, p < 0.001) (Figure S1E).

Although the bottom part of the cocoon appeared as a continuous layer of wax (Figure S1B) the upper part contained areas that were porous and transmitted light under the microscope. They did not contain pores of defined shape but they resembled a net where the wax layer was very thin or missing in certain areas (Figures S1C and S1D).

The respiration of the bumble bee pupae at the white eye stage was higher than in honeybees (median 7.68 μmol h^-^^1^ larva^-1^, n = 10) but that is not surprising given the bigger weight of the pupae (fresh weight median 0.345 g). When normalized to weight the respiration rate was remarkably similar (median 22.81 μmol g^−1^FW h^−1^, see Figure S1F and summary in Table S1). The area of the porous part of the bumble bee brood cocoon was larger in comparison to the area of the honeybee brood capping (Figure S1G) but its conductance was smaller. The calculated CO_2_ gradient across the cocoons made entirely from the porous material is 0.09%, similar to the 0.03% across honeybee brood cappings (Figure 5H). If the cocoon was made entirely from the compact waxy part the CO_2_ levels inside it could reach up to 11% (in case of calculating with maximal pupa respiration and minimal cocoon conductance values we recorded).

## Discussion

### Different cappings for different purposes

Bees have adapted to use wax in two contrasting biological ways: either as a nearly impermeable material insulating from the outside environment or as a permeable, porous material allowing the respiration and communication of developing pupa with its environment.

Similar but distinct evolutionary adaptations can be found in the wax usage of different *Hymenoptera*. While all the worker, drone and queen brood cappings of *A. mellifera* are intact, the drone brood cappings of *Apis cerana* and *Apis koschevnikovi* have a characteristic central opening.^27^^,^^28^ These openings serves for the exchange of respiratory gases by the developing drone pupae, as plugging them with beeswax leads to delayed metamorphosis or death.^29^ Moreover, the central openings allow the workers to smell easily any volatile signals emanating from the pupa. When a pupa is infected with the parasitic *Varroa* mite or by viruses, volatile signals instruct the worker bees to close the central opening in the capping by wax, entombing and killing both the pupa and the mite within the cell.^30^ Such hygienic behavior not only contributes to the varroa resistance of these species but it also puts a strong selective pressure on the mite to minimize the harm to the developing drone pupa.

In bumble bee colonies, the worker pupae develop in closed cells where the bottom part is made of wax but the upper part partially exposes the silky cocoon. Completely exposed cocoons are visible in the colonies of other social insect like wasps or ants (that neither raise brood nor store liquid food in wax cell-like structures within their colonies). Our data of bumble bee respiration and cocoon conductance suggest that the thin, porous layer of wax in the upper part of the cocoon allows sufficient gas exchange for the developing pupa. We conclude that bumble bees, similarly to honeybees, were able to evolutionary adapt to overcome the problem of low gas permeability of their waxy brood cells made of wax, by allowing some parts of it to be porous.

The honeybee brood cappings or the porous parts of the bumble bee cocoon represent a barrier against larger particles like grains of pollen that could potentially be a source of microbial contamination. However, despite the fact that the pores are large enough to allow entrance of individual bacteria or unicellular eukaryotes, it is unlikely that this way of entrance will play a major role in transmitting diseases. From the evolutionary point, we could speculate that capping of brood with wax was originally also beneficial by protecting the pupa from an attack of parasitic wasps, because it is common in some solitary bees such as the alpha-alpha leafcutting bees.^31^ It is obvious that protection against parasitic wasps does not play a role in the socially advanced honeybee or bumble bee colonies but this aspect might have been important at the beginning of eusociality evolution when colonies were small and not tightly organized. In relation to honeybees, the capping does not prevent the brood from being infected by the parasitic varroa mite, because the mite enters the cell before it gets capped. Nevertheless, it might be interesting to determine if the structure of the cappings, their variations in porosity, together with the accompanying changes in gas permeability affects mites physiological parameters, for example its fertility.

### The brood cappings have substantially increased gas permeability – Evolutionary convergences and physiological implications

We are the first to quantify both the honey and brood cappings CO_2_ conductances using a specialized tandem setup of Li-6400XT infra-red gas analyzers. We also used this up-to-date technique to measure the respiration of honeybee pupae and found remarkably similar values to the pupal respiration reported more than 80 years ago by *Melampy (1939)* using the Barcroft-Warburg manometric method^32^ (Table S1).

The pores of μm sizes that we identified in brood cell capping are responsible for the large increase in gas permeability of brood cappings in comparison to the solid wax of honey storage cappings. The pores in the brood cappings thus allow efficient exchange of volatile compounds, such as oxygen, carbon dioxide, pheromones or kairomones. As we demonstrate the CO_2_ concentration in the brood cells only mildly exceeds the CO_2_ concentrations in the hive (gradient of only 0.03%, so 300 ppm difference). This is important for proper brood development but also for the colony’s response to the pathogen infection.

It is remarkable that the conductance of the brood cappings for CO_2_ resembles the CO_2_ conductance of living cells, organs or surfaces, such as rat cardiomyocytes^33^ or human erythrocytes,^34^ despite being built from wax. The honey cappings have conductance on the opposite end of the biologically relevant spectrum, resembling the gas impermeable apical membrane of the gut cells^35^ (see also Figure 1C).

Another notable example of a convergent evolution can be seen in the plant aerial surfaces that are also covered with waxy cuticle. The cuticle is impermeable for gases and its conductance is close to values we measured for honey cell cappings.^36^ On the other hand, plants are able to substantially increase their conductance by means of closable pores – stomata. Strikingly, the percentage of brood capping area containing pores is similar to percentage of leaf area containing stomata.^37^ From this respect it may not be surprising that stomatous plant surfaces, such as the lower leaf sides, have similar conductances to the brood cappings.^38^

Although the CO_2_ conductance that we measured in the brood cappings is primarily the consequence of the pores, the low but not zero conductance of the honey capping is probably due to gas diffusion through the wax itself because of its partially crystalline structure^39^ or to an occasional pores that we rarely observed. It may be an interesting future challenge to separate CO_2_ flux into solid diffusion (wax) and air pathways using HelOx/NitOx techniques.^40^

### Estimated CO_2_ gradients and its consequences for brood development

Although it is obvious from our data that the brood cappings are well permeable to gases the honey cappings show very low and largely variable conductance. The conductivity data for honey cappings that we detected with the sensitive gas analyzers were close to the detection limit of the system and gave quite a wide range of small values close to zero, in a non-Gaussian distribution. Of interest, similar distribution of diffusive conductance was observed for intact plant cuticles.^41^ In addition, we found that the honey cappings were sensitive to handling. Some of the honey capping had unexpectedly high conductivity but inspection under a stereomicroscope revealed cracks that were obviously not native but rather caused by manipulation. Such obvious outliers were excluded from the analysis but cappings with less apparent faults might have been still included. It is therefore possible that despite the fact that the median conductance value was calculated to 3.3 mmol m^−2^ s^−1^, the real conductance of the honey cappings is lower, very close to zero. Such values would make the cappings virtually impermeable and the estimated CO_2_ gradient in a speculative situation of brood covered by honey cappings would far exceed the 15.2% maximal values we report.

Therefore, using honey cappings to cover brood would be very risky for the honeybees, as their workers are obviously not able to control the conductivity of this type of cappings and the CO_2_ levels in the brood cell could often rise beyond tolerable levels. Porous brood cappings, instead, seem to be perfectly elaborated and their conductance is more than sufficient and not deviating dramatically from Gaussianity.

Honeybees can tolerate a wide range of CO_2_ concentrations and the CO_2_ levels in the hive are maintained well above the 0.04% found in the free atmosphere. The usual CO_2_ concentrations in a beehive in summer fluctuate between 0.1 and 0.3%^42^^,^^43^ but up to 4% CO_2_ has been measured in small experimental hives in laboratory settings.^44^^,^^45^ Based on our data, the brood cappings are highly penetrable to CO_2_. Consequently, the CO_2_ concentration in a brood cell in the hive should lie within a narrow range of 0.13–0.33% for the majority of the season (Figure 5C).

Although the data on long term bee brood survival and physiological characteristics under elevated CO_2_ are missing in the literature, it is well documented that high CO_2_ exposure affects adult honeybee lifespan and interferes with their physiological functions.^46^^,^^47^^,^^48^ From this respect it is not surprising that honeybees are able to sense CO_2_ and its elevated levels and induce a fanning behavior to keep hive CO_2_ concentrations within tolerable limits.^44^

We used the *A. mellifera carnica* worker brood cappings and honey cappings to measure their chemical, physical and structural properties and found them similar to the properties of the bumble bee pupae cocoons of *Bombus terrestris*. It is therefore likely that similar principles apply to other *Hymenoptera* that use sealed cells for brood rearing or for storing their reserves. Sealing the wax cells brings an evolutionary advantage for these species and their ability to create wax structures with contrasting permeabilities represents an elegant evolutionary adaptation.

### Contrasting permeability of cappings as an evolutionary adaptation for using wax for nest building

When insects opted to use wax to cover their brood, they simultaneously needed to solve the issue of required gas permeability. However, whether this represented a real selection pressure or was merely reflected in the imperfect construction of such cappings *(i.e.* incorporating gas permeable pores or bare patches by default) is difficult to determine. Expressed another way, is it the cappings of brood or honey cells that represent the evolutionary adaptation? It is possible that initial imperfect cappings construction represented a default primary state that suited the respiratory requirements of covered brood, without the need of a selection. Accordingly, only later in their evolution did honeybees adapt this process to make the comparatively less permeable honey cappings, that in turn conveyed the evolutionary advantage of a long-term honey storage. However, although it is clear honey storage is an evolutionarily more recent development of the wax-building insects (as apparent from the bumble bee colonies that cover brood in wax but do not cap honey for long-term storage), it is still not easy to unequivocally determine whether it was the need for brood cappings permeability or honey cappings impermeability that represented the main selection pressure in the differential utilization of wax for these two types of cappings.

### Limitations of the study

A limitation of our study may be in our focus on *A. mellifera carnica* bees without consideration of the biological variability across honeybees in different environments, their subpopulations and geographical origins. Although similar values for capping conductance were measured across all the hives examined we cannot exclude the values may change or be specifically adapted by the bees during the worker brood developmental stages. Methodically, uncertainty in close-to-zero conductances of honey cappings is another limitation. Li-6400XT gas exchange system is very sensitive but developed to measure plant leaves of 2 × 3 cm (6 cm^2^). Honey or brood cappings have surface area about 0.2 cm^2^, compromising the absolute sensitivity. The positively skewed statistical distribution of honey capping conductance supports this idea. Moreover, the wax cappings are very sensitive to manipulation. Microscopic cracks appear easily, increasing gas permeability substantially. Minimal conductance of honey cappings may thus be even lower than we measured. This effect would further increase the differences between the honey and brood capping conductances and strengthen the significance of our study.

## STAR★Methods

### Key resources table

**Table:** 

REAGENT or RESOURCE	SOURCE	IDENTIFIER
**Biological samples**		

*Apis mellifera* carnica colonies	Experimental apiary of the Biology Centre AS CR, Ceske Budejovice, Czech Republic (48°58′31.924″N, 14°26′44.671″E; 390 m)	N/A
*Bombus terrestris* colony	Agricultural Science company in Troubsko, Czech Republic	http://www.ceskycmelak.cz/

**Chemicals, peptides, and recombinant proteins**		

C24 alkane	Sigma Aldrich	https://www.sigmaaldrich.com/CZ/en/product/sigma/t4758

**Software and algorithms**		

ImageJ	Schneider et al.^49^	https://imagej.nih.gov/ij/
STATISTICA 12	TIBCO Software Inc, Palo Alto, CA, USA	https://www.tibco.com/resources/

**Other**		

Gas chromatograph, Trace 1310	Thermo, Bremen, Germany	https://www.thermofisher.com/cz/en/home.html
Isotope ratio mass spectrometer (IRMS), Delta V Advantage	Thermo, Bremen, Germany	https://www.thermofisher.com/cz/en/home.html
Chromatography capillary column LION LN-05 Sil-MS	Chromservis, Prague, Czech Republic	https://www.chromservis.eu/en/lion-ln-5-sil-ms-gc-column-30-m-0-25-mm-0-10-m
6400XT infra-red gas analyser	LiCor, Nebrasca, USA	https://www.licor.com/env/products/photosynthesis/LI-6400XT/

### Resource availability

#### Lead contact

Further information and requests for resources and reagents should be directed to and will be fulfilled by the lead contact Alena Bruce Krejčí (abruce@prf.jcu.cz or akrejci@prf.jcu.cz) both addresses work.

#### Materials availability

This study did not generate new unique reagents.

### Experimental model and subject details

Colonies of *A. mellifera carnica* were kept at the experimental apiary of the Biology Center AS CR, Ceske Budejovice, Czech Republic (48°58′31.924″N, 14°26′44.671″E; 390 m) according to standard beekeeping protocol, including honey harvest in June and July, supplementary sugar feeding in July and August and varroa treatment with amitraz in the autumn and oxalic acid in winter. The levels of *Varroa destructor* were regularly monitored by alcohol washes and did not exceed 2% level during the whole season. Combs were built on wax foundations with 5.2 mm in short diagonal.

Bumble bee colony of *B. terrestris* was purchased in May 2022 from the company Agricultural Science in Troubsko, Czech Republic and placed at the same apiary as beehives described above.

### Method details

#### GC/MS analysis of waxes

Cappings were dissolved in chloroform (1 mg mL^−1^) and 2 μL of the sample were injected in split/splitless injector at 300°C of the GC (Trace 1310, Thermo, Bremen, Germany). Injection was splitless for 1.5 min, then split with flow of 100 mL per minute for the next 1 min, and 5 mL per minute (gas saver) for the rest of analysis. Fast column LION LN-05 Sil-MS (30 m × 0.25 mm x 0.1 μm film thickness) was used with helium flow rate 1.5 mL per minute. The oven temperature program was set to 50°C during injection and for next 2 min, then increasing to 200°C (slope 40°C/min), further to 320°C (4°C/min) and isothermal at 320°C for the rest of the analysis (*ca* 60 min in total). Eluting compounds were oxidized to CO_2_ via IsoLink II interphase (Thermo, Bremen, Germany) at 1000°C and introduced to continuous-flow isotope ratio MS (Delta V Advantage, Thermo, Bremen, Germany). Internal standard n-tetracosane (C24 alkane) was added to the samples in concentration of 20 μg mL^−1^ to quantify compounds. GC calibration curve for alkanes C10 to C40 was created to correct for any GC sensitivity drops for the high-boiling compounds. Principal compounds (alkanes and wax esters) were identified via available standards and/or literature available wax chromatograms.^11^^,^^12^^,^^21^^,^^22^^,^^23^^,^^24^ We also measured subset of derivatized samples (50 μL of BSTFA +100 μL of pyridine, 2 h at 80°C^25^) and found that overall peak area did not differ substantially (data not shown). The cappings also contain chloroform insoluble substances such as silk, pollen or hive debris but these were not quantified in our study. Five brood cappings and five honey cappings from the same comb were analyzed for 5 different honeybee colonies (n = 25 for brood cappings and n = 25 for honey cappings).

#### Measurement of the capping permeability to CO_2_

The honeybee brood cappings and honey cappings were carefully dissected from individual combs using scalpel and a pair of fine forceps in June 2022. Five cappings of both types were measured for each of 5 different hives (n = 25 for brood cappings and n = 25 for honey cappings). The CO_2_ diffusive conductance of the cappings was measured by a unique experimental system where two LI-6400XT units assembled together with their leaf chambers complemented in ‘inverse’ position (Figure 1B). One unit contained only the bottom part of chamber while the other unit contained only the upper part of chamber with an LED light source (not applicable here). A plexiglas plate 2 mm thick with a central hole of 3 mm in diameter was inserted between the chambers where plant leaf would normally be positioned. Dissected honey- or brood capping was sealed in the hole by pressing gently on its edge. The CO_2_ concentration in the upper unit was set to 2300 μmol mol^−1^ (maximal available concentration of the instrument) whereas lower unit was scrubbed of all CO_2_, producing a CO_2_ free air. The ‘matching conduct’ commonly present in the bottom part of the chamber was replaced with a needle and tubing. the setup was already optimized for measurement of amphistomatous leaves, where non only diffusional but also as bulk flow may occur across the leaf (stoma – intercellular air – stoma continuum). Accordingly, it was possible to monitor and maintain the pressure differences between the adaxial and abaxial unit less than 0.1 mBar (10 Pa) that is important to prevent a bulk flow across the pores.

The bumble bee brood cocoons were collected in June 2022 from a single colony of *B. terrestris*. Ten cocoons with white eyed pupae were carefully cut in their equatorial position and the upper (porous) and bottom (waxy) parts were measured separately using the tandem LI-6400XT system described above, with a thin parafin seal between the cocoon and the plexiglas in the measuring chamber.

#### Respiration rate measurement

Respiration of healthy honeybee worker brood pupae originating from 5 different colonies was examined at the stage of white to pink eyed pupae, 4 days post capping (10 days old since hatching). Five pupae were measured from each hive (n = 25). The bumble bee pupae of the white eye stage originated from a single colony of *B. terrestris*. The bumble bee brood respiration was measured in 10 intact cocoons and the stage of brood development was only examined after the cocoon dissection at the end of the experiment.

The rate of CO_2_ production of individual pupae was measured by Li-6400XT infra-red gas analyser with insect respiration chamber. The cappings of the brood cells were removed, respiring pupae were gently pulled out with entomological tweezers and maintained at 35°C throughout the measurements. The fresh weight (FW) and the dry weight (DW) of pupae was determined using microscales. Single pupae was inserted in the insect respiration chamber continuosly flushed with Li-6400XT sample CO_2_ free air stream of 150 μmol air s^−1^. After 5 min of stabilization, data were recorded for 3 min. We measured the average CO_2_ increase about 5 μmol mol^−1^, while the system noise was lower than 0.1 μmol mol^−1^, allowing us to determine the respiration rate with sufficient precision. As control, empty chamber was also measured regularly and any offset subtracted. The respiration rates were normalized both to FW or DW (see Table S1).

#### Microphotography, capped cell areas

Optical images of wax cappings were taken using Olympus BX61 microscope with a combination of transmitted and reflected light (to maximize pore to solid wax contrast) using objectives of 4x (frame 5.5 mm wide) and 10x (frame 2.2 mm wide). Sets of sequential images with focus stacking were merged into one picture with extended depth of field using the Combine ZP software. To precisely calculate the average area of cappings, individual unsealed honey comb cells were photographed (n = 18) under a stereomicroscope with a reference scale and their inner area calculated with ImageJ Software.^26^ Individual halves of the bumble bee cocoons (n = 11) were also photographed and equatorial diameter was used to calculate surface area approximated as a hemisphere + cylinder 3 mm in height.

Individual pore area, total pore area per brood capping and median pore diameter were quantified in ImageJ using microphotographs of cappings originating from 7 different hives, with 5 cappings measured for each hive (n = 35).

#### Scanning electron microscopy

For scanning electron microscopy (SEM), samples were coated by gold (2 nm thin layer) in an ion sputter coater (Bal-Tec SCD 050) and observed with a JEOL JSM-IT 200 microscope.

### Quantification and statistical analysis

Statistics was performed in the *Statistica 12* software (TIBCO Software Inc, Palo Alto, CA, USA). Wax composition (Figure 2B) and bee-pupae respiration (Figure 5A) had a Gaussian within-group distributions (Shapiro-Willk test) and therefore they were tested for differences using ANOVA. Wax composition was tested by 3-way full factorial ANOVA(fixed factors: ‘compound’, ‘capping type’; random factor: ‘hive’). Pupae respiration rates were tested by 1-way ANOVA(factor: ‘hive’). Capping diffusive conductances (Figures 1A and S1), in turn, had positively skewed distribution, mainly in group of ‘honey cappings’, and therfore they were tested by Mann-Whitney U test (factor: ‘capping type’). Graphically, raw data distributions, rather than descriptive statistics, are presented. Significance was defined as p < 0.001 (∗∗∗), 0.001 < p < 0.01(∗∗), 0.01 < p < 0.05 (∗), p > 0.05 (ns). The sampling and number of replicates are indicated in the specific sections of the STAR methods and in the figure legends.

## Data Availability

• All data reported in this paper will be shared by the lead contact upon request. • This paper does not report original code. • Any additional information required to reanalyze the data reported in this paper is available from the lead contact upon request.
