# Supplementary material for: Honeybees control the gas permeability of brood and honey cappings
Source: iScience. 2022 Oct 27;25(11):105445. doi: 10.1016/j.isci.2022.105445 (PMC9650039; doi:10.1016/j.isci.2022.105445)
Supplement: Document S1. Figure S1 and Table S1 [file mmc1.pdf]

iScience, Volume *25*

Supplemental information

Honeybees control the gas permeability of brood and honey cappings

Jiří Kubásek, Karolína Svobodová, František Půta, and Alena Bruce Krejčí

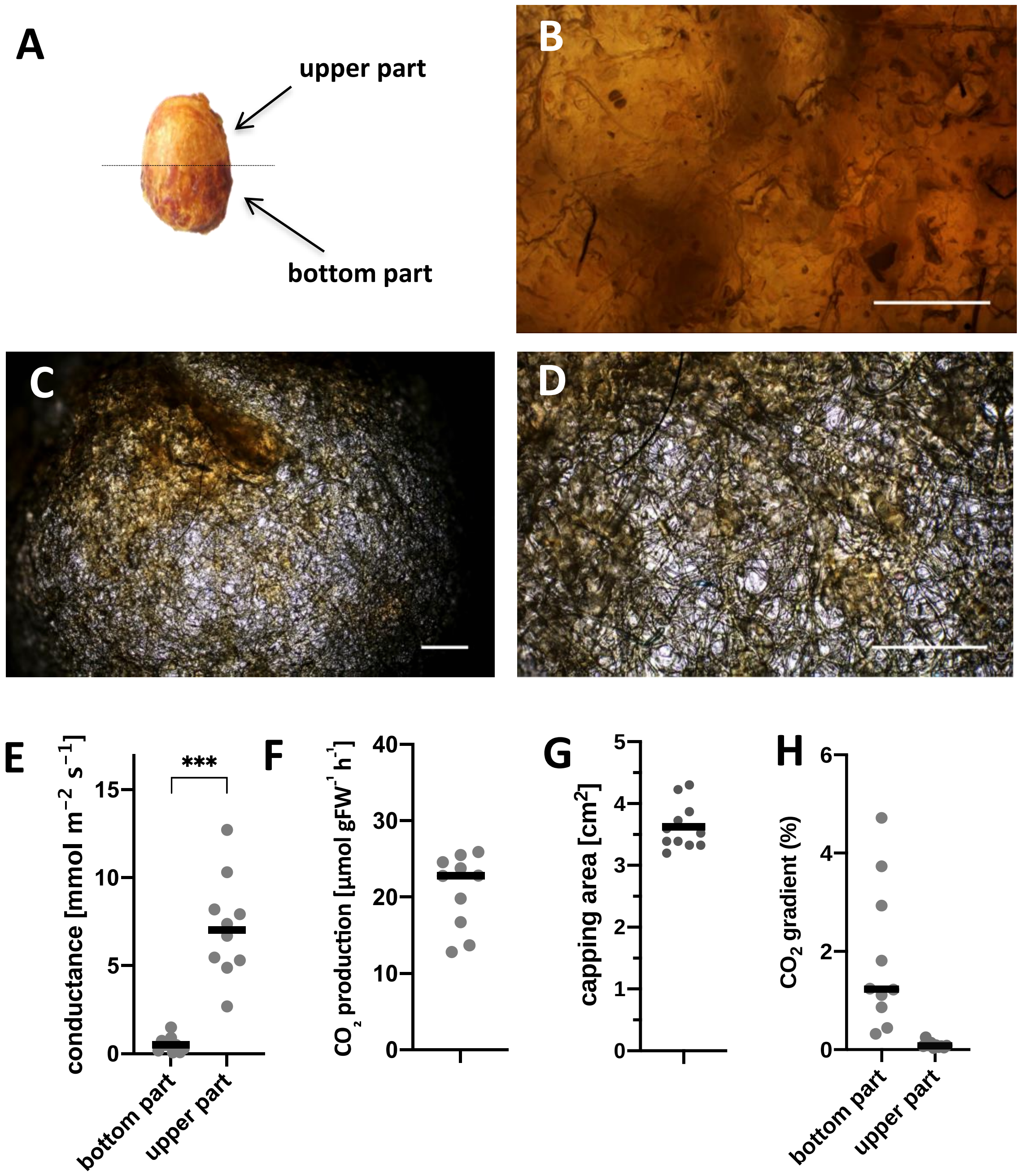

Suppelmentary figure S1: The conductace of bumble bee brood cocoons (Bombus terrestris), respiration of their pupae and expected CO_2 gradient across the cocoon. Related to Fig. 1, 3 and 5. (A) The cocoon consists of bottom waxy part and upper porous part. (B) Microphotography of the bottom waxy part. Scale bar 500 μ m. (C, D) Microphotography of the upper porous part. Scale bar 500 μ m. (E) The conductance of the upper and bottom part of the cocoon (n=10). Mann-Whitney U test (U=0, Z= -3.74, p<0.001) (F) CO2 production of the white eyed pupae (n=10). (G) The area of the porous half of the cocoon (n=10). (H) Calculated CO2 gradients across the cocoons in case they were made entirely from the material forming the bottom or upper part (n=10). E-F: Individual values with median. See also Supplementary table S1.

	Age of pupae	Method	CO_2 produced	CO_2 produced	fresh weight	respiration rate FW	respiration rate FW	dry weight	respiration rate DW	respiration rate DW
	days		(µmol h-1 per pupa)	(mm-3 h-1 per pupa)	(g)	(µmol gWW-1 h-1)	(mm-3 gFW-1 h -1)	(g)	(µmol gDW-1 h-1)	(mm-3 gDW-1 h -1)
Apis mellifera (this study)	10	Li-6400XT	2.37	53.02	0.1445	16.39	367.14	0.0296	79.92	1790.21
Apis mellifera (Melampy,1939)	10	Barcroft-Warburg	2.42	54.1	0.1356	17.81	399.00	-	-	-
Bombus terrestris (this study)	-	Li-6400XT	7.13	159.63	0.3479	20.85	533.08	0.10103	71.37	1767.93

Supplementary table S1: The overview of values for honey bee and bumble bee pupae weight and respiration. Relates to Fig. 5 and S1.
